# Pan-Tropical Analysis of Climate Effects on Seasonal Tree Growth

**DOI:** 10.1371/journal.pone.0092337

**Published:** 2014-03-26

**Authors:** Fabien Wagner, Vivien Rossi, Mélaine Aubry-Kientz, Damien Bonal, Helmut Dalitz, Robert Gliniars, Clément Stahl, Antonio Trabucco, Bruno Hérault

**Affiliations:** 1 Remote Sensing Division, National Institute for Space Research - INPE, São José dos Campos, SP, Brazil; 2 Cirad, UMR 93 “Ecologie des Forêts de Guyane,” Kourou, France; 3 Cirad, UR 105 “Biens et services des écosystèmes forestiers tropicaux,” Montpellier, France; 4 Université de Yaoundé 1, UMI 209 “Modélisation Mathématique et Informatique de Systèmes Complexes,” Yaoundé, Cameroun; 5 Université des Antilles et de la Guyane, UMR 93 “Ecologie des Forêts de Guyane,” Kourou, France; 6 INRA, UMR EEF 1137, Champenoux, France; 7 Institute of Botany, University of Hohenheim, Stuttgart, Germany; 8 CIRAD, UMR “Systèmes d'Elevage en Milieux Méditerranéens et Tropicaux,” Kourou, France; 9 Euro-Mediterranean Centre for Climate Change, Sassari, Italy; 10 Division of Forest, Nature, and Landscape, KU Leuven, Leuven, Belgium; Bangor University, United Kingdom

## Abstract

Climate models predict a range of changes in tropical forest regions, including increased average temperatures, decreased total precipitation, reduced soil moisture and alterations in seasonal climate variations. These changes are directly related to the increase in anthropogenic greenhouse gas concentrations, primarily CO_2_. Assessing seasonal forest growth responses to climate is of utmost importance because woody tissues, produced by photosynthesis from atmospheric CO_2_, water and light, constitute the main component of carbon sequestration in the forest ecosystem. In this paper, we combine intra-annual tree growth measurements from published tree growth data and the corresponding monthly climate data for 25 pan-tropical forest sites. This meta-analysis is designed to find the shared climate drivers of tree growth and their relative importance across pan-tropical forests in order to improve carbon uptake models in a global change context. Tree growth reveals significant intra-annual seasonality at seasonally dry sites or in wet tropical forests. Of the overall variation in tree growth, 28.7% was explained by the site effect, i.e. the tree growth average per site. The best predictive model included four climate variables: precipitation, solar radiation (estimated with extrasolar radiation reaching the atmosphere), temperature amplitude and relative soil water content. This model explained more than 50% of the tree growth variations across tropical forests. Precipitation and solar radiation are the main seasonal drivers of tree growth, causing 19.8% and 16.3% of the tree growth variations. Both have a significant positive association with tree growth. These findings suggest that forest productivity due to tropical tree growth will be reduced in the future if climate extremes, such as droughts, become more frequent.

## Introduction

Tropical forests are being threatened on an unprecedented scale by global changes. Temperatures across tropical forest regions are currently increasing [1] and are expected to continue to increase with a concomitant decrease in precipitation over the next decades [2]–[4]. Climate models predict a range of changes in tropical forest regions, including increased frequency of extreme climatic events, increased average temperatures, increased atmospheric CO_2_ and changes in seasonal distribution and interannual variability of rainfall [5]–[9]. Tropical forests play an important role in the mitigation of anthropogenic atmospheric CO_2_ emissions by constituting a major reservoir of terrestrial carbon and a large and persistent carbon sink [10]–[12]. Feedback between tropical forests and the local and regional climate has also been demonstrated [4], [13].

Tree growth is linked with atmospheric CO_2_ through photosynthesis. The last 20 years have seen a substantial increase in the number of publications focusing on the effects of climate on tropical tree growth. A search performed on Web of Science in March 2013 using the keywords 'climate', 'tropical forest', 'growth' and 'trees' returned fewer than 15 articles per year before 2000 and more than 60 articles in 2012, for a total of 541 articles focusing on the effect of climate on tropical tree growth. Long-term variations in tree growth have been reported on long-term forest plots, but the determinants of these variations are still being discussed [14]–[19]. Due to the annual or multi-annual census frequency of long-term forest plots, most studies focus on the annual or multi-annual variation in tree growth even though most tropical forests undergo an intrannual seasonality in climate [1], [6], [20]–[22]. In single-site-based studies, seasonal rhythms of tree growth have been linked to seasonal variations in water availability, rainfall, temperature and solar radiation (Table 1).

**Table 1:** Expected tropical tree growth response to climate variables.

variable	predicted effect^a^	references	process*^b^*
*REW*	+	[28], [62]	photosynthesis, xylem tension, stomatal closure, leaf flush
			
*rainfall*	+	[19, 23, 24, 35, 58, 59, 61, 97–90]	photosynthesis, xylem tension, stomatal closure, leaf flush
	−	[89], [91]	
*T mean*	−	[31]–[33], [92], [93]	photosynthesis kinetic, stomatal closure
*T min*	−	[14], [17], [35], [66]	photosynthesis kinetic, stomatal closure
	no	[94], [95]	
*T max*	−	[19], [31], [33]	photosynthesis kinetic, stomatal closure
	+	[96]	
	no	[94], [95]	
*VPD*	no	[97], [98]	stomatal closure, transpiration
*irradiance*	+	[17], [21], [29], [30], [64], [70], [74]	photosynthesis, phenology
	−	[21]	
	no	[17], [63], [70]	
U*	+	[99]	photosynthesis, transpiration

a : expected growth response to the climate variable: (+) trees are expected to grow faster with high values of the climate variable, (−) trees are expected to grow slower with high values of the climate variable. *^b^*: biological processes involve in the tree growth response to a given climate variation. *VPD* is vapour pressure deficit, and Friction velocity (U*) is a climate variable provided by eddy flux data, which is correlated with wind speed. Relative extractable water (REW), is a daily value between 0 and 1; when 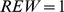, the amount of extractable water by the tree is at its maximum and when 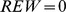, no water is available for the trees [28].

Rain or lack of rain is often implicitly viewed as the main drivers of forest dynamics [23], as annual net primary production (NPP) positively correlates with the annual sum of precipitation at large scales [24] and rainfall seasonality plays a key role in the forest's response to climate variability [25]. The relation between the amount of rainfall and water availability for trees is not straightforward and is determined by various soil and plant characteristics (i.e. permanent wilting point, field capacity, root distribution). Consequently, water stresses are increasingly estimated using soil water balance models [26], [27], including some that are explicitly designed for tropical forests [28]. Irradiance is directly linked to plant photosynthetic capacity, which in turn drives carbon uptake and plant growth [29]. The occurrence of dry periods linked to cloud-cover reduction was found to enhance canopy photosynthetic capacity by 25% throughout Amazonia [30]. The effects of rising temperatures on the physiology of tropical trees are currently debated within the scientific community [21], [32]. Some studies suggest that reductions in photosynthetic rates at temperatures above 30°C are driven by reductions in stomatal conductance in response to higher leaf-to-air vapour pressure deficits [31] or by a direct down-regulation of biochemical processes during CO_2_ fixation [33,33]. Recent studies, however, suggest that tropical tree mortality may increase significantly with increasing night-time temperatures, while tree growth appears surprisingly sensitive to variations in mean annual night-time temperatures of 1–2°C [35].

A pantropical analysis of the effects of climate seasonality on tropical tree growth is still missing in the literature. Most of our knowledge comes from single-site-based studies that often suffer from collinearity problems between climate drivers. In this paper, we ran a meta-analysis of monthly tropical tree growth at the pan-tropical scale in which multiple gradients of climate variables allowed us to disentangle the effect of each climate driver on tropical tree growth. We focused on the seasonal effect of climate on tree growth in tropical forests by using data from papers reporting tree growth measurements with a high periodicity (from daily to monthly censuses) and global climate datasets. We gathered 30 datasets in which growth measurements have been recorded for 3412 individual trees from 25 pantropical forest sites. This paper has three specific objectives: (i) to find the climate drivers of tree growth across tropical forests; (ii) to quantify tree growth variations in response to climate among tropical forests; and (iii) to give a modeling framework to improve the model predictions of seasonal carbon uptake by tropical tree growth in a global change context.

## Materials and Methods

### Climate datasets

We used climate datasets from two sources (Table 2): the Climate Research Unit (CRU) at the University of East Anglia [36] and the Consortium for Spatial Information website (CGIAR-CSI, http://www.cgiar-csi.org). From the CRU, we used variables from the CRU-TS3.1 and CRU-TS3.10.01 monthly climate global datasets available at 0.5° resolution from 1901–2009: cloud cover (*cld*, unit:%); precipitation (*pre*, mm); daily mean, minimal and maximal temperatures (respectively *tmp*, *tmn* and *tmx*, °C); temperature amplitude (*dtr*, °C); vapour pressure (*vap*, hPa); and potential evapotranspiration (*pet*, mm). *pet* was calculated using the grass reference evapotranspiration equation [37], [38], which is a variant of the Penman-Monteith method using the gridded *tmp*, *tmn*, *tmx*, *vap* and *cld*. *pre* was square-root transformed prior to data modeling to address heteroscedasticity. From the CGIAR-CSI, we used the monthly average of extraterrestrial solar radiation as well as the Global Soil-Water Balance [27]. The first dataset defines the solar radiation reaching the top of the Earth's atmosphere (*sol*) and is a function of Earth-sun geometry and time of year. *sol* per day (mm/day equivalent) is calculated using the methodology presented in [39] specifically for the 15*^th^* day of each month to describe averages per month. Total *sol* per month (mm/month equivalent) is calculated by multiplying the value of *sol* for the 15th day of the month by the number of days in the month, 1 mm.day^−1^ equivalent of evaporation  =  2.45 MJ.m^−2^.day^−1^. The latter dataset provides hydrological raster data (ESRI Grid format) describing actual evapo-transpiration and soil water content for a monthly time-series from period 1901–2009 using CRU-TS3.1 as the primary climate data input. The monthly time-series of relative soil water content *swc* (unitless, 0–1) is calculated as the ratio of the soil water content from the soil water balance over the maximum available soil moisture (in mm water per 1 m soil depth, from the Digital Soil Map of the World and Derived Soil Properties [40], [41]) along the ecosystem rooting depth [42].

**Table 2:** Climate datasets used to model seasonal tropical tree growth at a pantropical scale.

climate variable	full name	unit	origin
*pre*	precipitation	mm	CRU-TS3.10.01
*sol* a	Extraterrestrial solar radiation	mm/month as equivalent of evaporation	CGIAR-CSI
*cld*	cloud cover	%	CRU-TS3.1
*Pre*	Potential Evapotranspiration	mm	CRU-TS3.1
*swc*	relative soil water content	unit less	CGIAR-CSI
*tmp*	mean temperature	°C	CRU-TS3.1
*tmn*	minimal temperature	°C	CRU-TS3.1
*tmx*	maximal temperature	°C	CRU-TS3.1
*vap*	vapour pressure	hPa	CRU-TS3.1
*dtr*	temperature amplitude	°C	CRU-TS3.1

a : *sol* per day (mm/day equivalent) is calculated using the methodology presented in [39] specifically for the 15*^th^* day of each month to describe averages per month. Total *sol* per month (mm/month equivalent) is calculated by multiplying the value of sol for the 15th day of the month by the number of days in the month, 1 mm.day^−1^ equivalent of evaporation  =  2.45 MJ.m^−2^.day^−1^.

### Tree growth data

**Figure 1:**
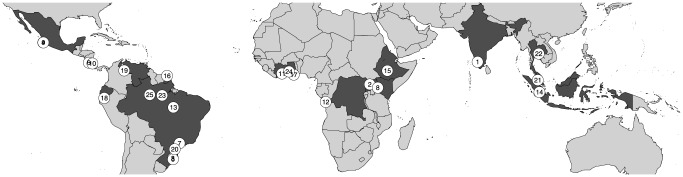
Locations of the 25 study sites and their countries (grey areas). 1: Attapadi; 2: Budongo; 3: CPM; 4: El Palmar; 5: FLONA SFP; 6: Guanacaste; 7: Ibicatu; 8: Kakamega; 9: La Barcinera; 10: La Selva; 11: Lamto; 12: Luki forest; 13: Marajoara; 14: Muara Bungo; 15: Munessa-Shashamene Forest; 16: Paracou; 17: Pinkwae; 18: RBSF; 19: RFC; 20: Rio Cachoiera; 21: Selangor plantation; 22: SERS; 23: Tapajos; 24: Tinte Bepo; 25: ZF-2.

A search performed on Web of Science in March 2013 using the keywords 'climate', 'tropical forest', 'growth' and 'trees' returned 541 articles focusing on effect of climate on tropical tree growth. Among these publications, our analysis used only the publications with seasonal tree growth data (from daily to monthly censuses) available in the article graphics, available in an online repository or provided by the authors. The tree growth data were extracted from the article graphics using WebPlotDigitizer 2.5 (http://arohatgi.info/WebPlotDigitizer/, Table 3). Three types of data were found: (i) single observations by tree and time step at a given site (see [43]); (ii) mean and standard deviation (SD) by species at a given site for each time step (see [44]); and (iii) only the mean and SD of all trees at a site for each time step [35]. In addition, these data may come from measurements with dendrometer bands, electronic point surveys, tree ring analyses or graduated tapes. The minimum diameter at breast height (DBH) used in the articles is 10 cm. Among sites, Selangor and Muara Bungo are forest plantations. Finally, they have different time step between measurements: monthly, weekly or daily. We converted these primary datasets by making a linear approximation of the growing trajectory in order to obtain a mean and a standard deviation by month and dataset for each available calendar year. Datasets from the same site were assumed to be samples from the same population and were not analyzed separately. This could affect the value of the whole population mean growth, but here we are only interested in the monthly variations of tree growth (site effect is considered as a random effect). The final dataset is comprised of 30 datasets of 25 pantropical forest sites, for a total of 3412 individual trees (Figure 1, Table 3). The data, monthly tree growth mean and SD, are freely available upon request to the corresponding author. No specific permits were required for the described field studies and this study did not involve endangered or protected species.

**Table 3:** Description of the tree growth data.

reference	country	site	Long.	Lat.	method	time scale	No. of trees	Type*^b^*	duration (mm/yyyy)	dbh growth mean±SD (mm/month)
[78]	Ghana	Tinte Bepo	−2.10	7.07	dendrometer band	monthly	42	2	11/1997–3/1999	0.32 ± 0.28
[43]	Costa Rica	Guanacaste	−85.50	10.75	dendrometer band	daily	19	3	2/1978–1/1979	0.27 ± 0.33
[100]	Ecuador	RBSF	−79.07	−3.97	electronic point dendrometer	daily	5	3	4/2006–2/2009	0.27 ± 0.38
[101]	Brazil	Rio Cachoiera	−49.71	−25.25	dendrometer band	weekly	120	2	11/2007–9/2008	1.11 ± 0.97
[35]	Costa Rica	La Selva	−84.00	10.43	dendrometer band	monthly	28	1	6/2006–11/2008	0.3 ± 0.09
[44]	DRC	Luki forest	13.18	−5.58	graduated tape	monthly	30	2	5/2006–7/2007	0.19 ± 0.59
[102]	Brazil	ZF-2	−60.12	−2.59	dendrometer band	Monthly	272	1	1/2000–12/2000	0.15 ± 0.05
[103]	Costa Rica	Guanacaste	−85.50	10.75	dendrometer band	weekly	40	3	8/1969–7/1970	0.39 ± 0.37
[104]	French Guiana	Paracou	−52.91	5.28	dendrometer band	weekly	9	3	10/1979–6/1982	0.64 ± 0.23
[105]	Ivory Coast	Lamto	−5.03	6.22	dendrometer band	daily	6	3	2/1973–7/1981	0.28 ± 0.2
[106]	Brazil	Tapajos	−54.97	−2.85	dendrometer band	monthly	734	3	12/2001–12/2005	0.31 ± 0.14
[107]	Uganda	Budongo	31.54	1.73	dendrometer band	monthly	318	3	7/2003–12/2009	0.1 ± 0.05
[19]	Kenya	Kakamega	34.86	0.35	dendrometer band	monthly	770	3	7/2003–12/2009	0.19 ± 0.03
[90]	Brazil	Marajoara	−50.27	−7.83	dendrometer band	monthly	67	2	2/1997–11/2001	0.5 ± 0.55
[90]	Brazil	Marajoara	−50.27	−7.83	dendrometer band	monthly	40	3	1/1997–10/2001	0.51 ± 0.55
[80]	Ethiopia	Munessa-Shashamene Forest	38.87	7.43	electronic point dendrometer	daily	4	3	4/2008–8/2009	0.45 ± 0.39
[108]	Ghana	Pinkwae	−0.13	5.75	dendrometer band	weekly	79	2	2/1978–4/1979	−0.2 ± 0.78
[108]	Ghana	Pinkwae	−0.13	5.75	dendrometer band	weekly	2	3	3/1978–4/1979	−0.24 ± 0.79
[109]	Brazil	Ibicatu	−47.72	−22.78	dendrometer band	monthly	5	3	3/1999–4/2006	0.34 ± 0.47
[110]	Mexico	El Palmar	−104.47	19.13	dendrometer band	monthly	23	2	9/2002–8/2003	0.24 ± 0.36
[110]	Mexico	La Barcinera	−104.42	19.15	dendrometer band	monthly	14	2	9/2002–8/2003	0.09 ± 0.29
[61]	Brazil	Tapajos	−54.97	−2.85	dendrometer band	monthly	450	2	11/1999–5/2001	0.21 ± 0.06
[111]	Malaysia	Selangor plantation	101.52	3.51	dendrometer band	weekly	6	3	1/1993–12/1995	0.55 ± 0.32
[112]	Thailand	SERS	101.93	14.50	dendrometer band	weekly	6	3	4/2004–2/2006	0.04 ± 0.04
[113]	Brazil	CPM	−50.50	−29.00	tree ring analysis	monthly	12	1	5/2005–5/2006	0.08 ± 0.08
[114]	India	Attapadi	76.45	11.08	dendrometer band	monthly	101	3	3/1980–10/1983	0.19 ± 0.12
[79]	French Guiana	Paracou	−52.91	5.28	dendrometer bands	weekly	161	3	6/2007–1/2009	0.14 ± 0.06
[115]	Indonesia	Muara Bungo	102.21	−1.49	meter	monthly	40	3	4/2004–4/2006	0.84 ± 0.23
[116]	Ecuador	RBSF	−79.07	−3.97	electronic point dendrometer	daily	1	3	4/2006–8/2009	0.23 ± 0.38
[117]	Venezuela	RFC	−70.75	7.50	dendrometer band	monthly	6	3	4/1978–4/1982	0.75 ± 0.52
[118]	Brazil	FLONA SFP	−50.42	−29.42	dendrometer band	monthly	2	3	9/2003–7/2006	0.23 ± 0.19

Description of the data used in this analysis, references, site location, site name, latitude and longitude in °, method of measurement, number of trees, follow-up type (*^b^*: Type 1: only mean and sd for the N trees of a site per month, Type 2: mean and sd by species for a site per month and Type 3: one observation by tree for a site per month.), follow-up duration and mean ± standard deviation of monthly diameter at breast height (dbh) growth.

### Preliminary analysis

First, we investigated the association between climate variables on a monthly time scale through a principal component analysis (PCA) on the normalized climate dataset, i.e. climate variables were centred and scaled, to describe how the variance of the climate dataset was structured. Next, to measure spatial autocorrelation in tree growth observations between sites, we computed the Moran's Index statistics of the mean monthly growth by sites [45]. This index range from -1, strong negative spatial autocorrelation, to +1, strong positive spatial autocorrelation. The significance of Moran's I is evaluated by using a Z score and p-value generated by random permutation [46]. The null hypothesis states that there is no spatial autocorrelation for the variable within the geographic area. The interannual and intra-annual variability climate variable was described by computing the coefficient of variation (CV) for each variable (i.e. standard deviation × 100 divided by the mean) of the annual mean and monthly values of the climate time series over the period of the CRU data (1901–2009). To detect, estimate and test seasonal patterns in the tree growth time series, we used temporal regression models from the R package season [47]. The model was fitted using a sine and cosine term that together described the sinusoid. These parameters were added to a generalized linear model to explain tree growth data and test the existence of a seasonal pattern. The existence of a seasonal pattern was determined by the zero-test based on Snedecor's F statistic. This method is known as the cosinor test.

### Modeling seasonal tree growth

We modeled the link between tree growth, site and climate variables in a mixed linear model framework at a monthly time step. Here, we assumed that each growth measurement was independent and that our multiple-site design enabled us to deal with the collinearity of climate variable present in each site. We set the climate variables as fixed effects and the site as a random effect. We set the site effect as a random effect in order to avoid any statistical bias in our results. This site effect was not further analyzed as the main objective of the study was to understand seasonal variations of tree growth. In order to rank the climate effects, we considered various growth models:

• 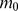, the reference model:
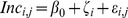
(1)


•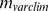, for each climate variable 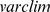, an univariate model:
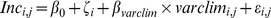
(2)


•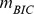, the model with the best combination of climate variables, *Comb_BIC_*, according to the Bayesian Information Criterion (BIC):
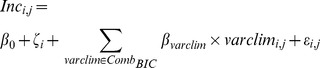
(3)


Where 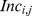 is the average tree diameter growth for the site 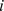 for the month 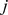, 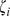 is the random site effect assumed to follow a normal distribution 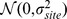 and 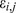 is the residual error assumed to follow a normal distribution 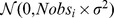 weighted by 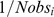, which is the inverse of the number of observation periods by site. The mixed model parameters were estimated with the algorithms of the R package lme4 [48]. To estimate the parameter confidence intervals with a probability of 95%, we generated 10000 parameter samples from the posterior distribution of the fitted model parameters using the Markov Chain Monte Carlo methods [48]. Then, the confidence intervals for the parameters (Highest Posterior Density [HPD] intervals) were constructed from the empirical posterior distribution of the 10000 samples as the intervals containing the parameters with the nominal probability [48]. Evaluation of the model performance was made by computing goodness-of-fit-measures with the R package lmmfit [49]–[52]. To find the best variable linear combination that contains the maximum of information to link growth and climate variables (the model *m_BIC_*), we ran an exhaustive screening of the candidate models using a stepwise procedure based on the Bayesian information criterion, BIC [53]. We used BIC, instead of the classically used AIC, to avoid over-parameterization and multicollinearity problems, as this criterion is consistent and parsimonious for model selection with respect to large datasets [54]. We made a residual analysis to verify if the error of the model *m_BIC_* had a bias for any of the selected variables in this model. That is, we computed the Pearson's product-moment correlation coeffiecient (*ρ*) between the residual of the model *m_BIC_* and the variables of this model and tested if this coefficient was statistically or different not from zero.

The predictive quality of the fitted models was assessed by computing the root mean square errors of predictions, RMSEP.
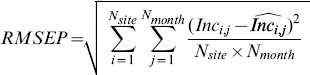
(4)


where 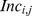 is the observed values of tree growth for the site *i* and for the month *j*, and 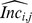 are the model predictions of growth.

All analyses were performed using the R-project software (http://www.r-project.org/).

## Results

### Climate gradients

The 25 sites represent a large sample of tropical forests under different tropical climates corresponding to five global ecological tropical zones [55](Table 4). The gradient of the annual mean of precipitation ranges from 973.9 mm (RBSF, Ecuador) per year to 3948.1 mm (La Selva, Costa Rica). The coefficient of variation (CV) of interannual precipitation ranges from 12.5% (SERS, Thailand) to 35.2% (RBSF, Ecuador). Intra-annual CV of monthly precipitation distribution precipitation ranges from 13% (CPM,Brazil) to 116.9% (El Palmar and La Barcinera, Mexico). The sites undergo a large variability in the distribution of precipitations throughout the year, from zero months (La Selva) to 8–9 months with less than 100 mm of rain (Lamto, Tinte Bepo, Pinkwae, RBSF). The relative soil water content exhibits the same intra-annual pattern, ranging from sites without seasonality, intra-annual CV*_swc_* < 3.5% (Cpm, La Selva, Muara Bungo, Rio Cachoiera and Selangor), to sites with an intra-annual CV*_swc_* > 37.8% (El Palmar, la Barcinera, RBSF). The annual average of relative soil water content is above 60% for 24 of the 25 sites. The annual cloud cover mean ranges from 42.9% (Munessa-Shashamene, Ethiopia) to 87.2% (Luki Forest, DRC) and its intra-annual variation is similar to the intra-annual variation of precipitation.

**Table 4:** Descriptive statistics of climate variables.

site	GES*^a^*	pre	pet	tmn	tmp	tmx	dtr	swc	vap	cld	sol
Attapadi	TAr	2158 (18.1/100.5)	1498.3 (1.9/19.9)	21.4 (2.1/5.8)	25.7 (1.8/5.2)	30.2 (1.7/6.2)	8.8 (3.8/19.6)	0.8 (3.4/30.6)	24.7 (2.5/9.3)	50.5 (2.5/38.2)	5214.7 (0/7.7)
Budongo	TAr	1322.1 (15.4/41.6)	1363.8 (2.8/10)	17.5 (4/3.1)	23.4 (2.9/3.5)	29.3 (2.4/4.4)	11.9 (3/9)	0.8 (8/20.2)	20.5 (4.2/5.5)	59.9 (3.5/9.3)	5313.8 (0/3.3)
CPM	SCf	1741.4 (16.9/13)	935.1 (2.4/34.2)	12.1 (4/27.4)	16.5 (2.6/20.6)	21 (1.9/16.8)	9 (2.4/5.1)	1 (2.3/1.5)	15.3 (3.3/21.4)	65.8 (2.7/5.9)	4745 (0/28.4)
El Palmar	TAwa	1076.3 (26.8/116.9)	1414.3 (2.4/12.9)	17.7 (2.6/16.7)	24.6 (1.6/7.1)	31.6 (1.4/4)	13.9 (3.3/20.5)	0.6 (9.1/42.1)	21 (1.8/15)	55.5 (4.7/22.9)	5028.8 (0/14.5)
FLONA SFP	SCf	1643.1 (16.2/13.6)	888.7 (2.6/32.7)	11.6 (4.1/28.2)	15.9 (2.7/20.7)	20.2 (1.9/16.4)	8.6 (2.3/3.4)	0.9 (4.1/4.7)	15 (3.4/21.5)	66.8 (2.5/6.5)	4729.1 (0/28.8)
Guanacaste	TAwb	1749.7 (24/86.8)	1606.9 (2.8/19)	21.1 (2.7/3.3)	26.8 (2.1/3.4)	32.5 (1.7/4.1)	11.4 (0.6/9.8)	0.9 (5.1/20.2)	28.2 (2.7/6.5)	49.9 (2/20.1)	5220.8 (0/7.4)
Ibicatu	TAwa	1518.2 (22/59.8)	1097.7 (2.5/18.9)	16.1 (5/17.3)	21.3 (3.4/11)	26.6 (2.6/7.4)	10.5 (3.8/8.8)	0.9 (10.1/14.3)	18.2 (3.9/18.1)	73.7 (0.9/10.1)	4966.1 (0/22.3)
Kakamega	TM	1770.9 (14.4/37)	1501.1 (2.7/13.3)	14 (4.3/3.9)	21.4 (2.7/3.2)	28.8 (2.2/4.1)	14.9 (2.8/8.1)	0.9 (7/15.7)	15.5 (3.5/6.5)	62.9 (3.6/6.3)	5317.5 (0/3.8)
La Barcinera	TAwa	1076.3 (26.8/116.9)	1414.3 (2.4/12.9)	17.7 (2.6/16.7)	24.6 (1.6/7.1)	31.6 (1.4/4)	13.9 (3.3/20.5)	0.6 (11.5/40.2)	21 (1.8/15)	55.5 (4.7/22.9)	5028.8 (0/14.5)
La Selva	TAr	3948.1 (19.4/44.7)	1358.2 (3.7/13.5)	17.9 (3.9/3.4)	23.1 (3/2.6)	28.3 (2.4/3.4)	10.4 (0.6/10.3)	1 (1.7/2.9)	23.5 (3.4/5)	52.9 (2.9/13.1)	5225.8 (0/7.2)
Lamto	TAr	1381.5 (21.3/60.9)	1122.5 (1.5/15.4)	21.8 (1.5/3.7)	26.5 (1.2/3.7)	31.2 (1.1/5.3)	9.4 (2.5/18.1)	0.9 (5.7/16.5)	27.3 (2/4.8)	77.2 (1.1/15.8)	5282.3 (0/4.3)
Luki forest	TAr	1069.1 (18.4/83.7)	947.8 (1.3/12.8)	20.8 (1.3/8.9)	24.6 (1.1/8.5)	28.6 (1/8.2)	7.8 (0/7.8)	0.8 (6.3/17.1)	24.8 (1.7/11.6)	87.2 (0.8/5.9)	5301.4 (0/7.6)
Marajoara	TAr	1873.6 (12.8/74.2)	1068.4 (2.6/14.9)	19.5 (3.5/4.8)	25.6 (2.6/1.7)	31.8 (2.1/4.3)	12.3 (0/17.5)	0.8 (4.5/21.6)	28.5 (3/4.1)	77.2 (0.2/20.6)	5281.7 (0/9.4)
Muara Bungo	TAr	2686.8 (14.6/34.2)	1260.5 (2.9/4.7)	22.9 (2/1.6)	27.2 (1.7/1.3)	31.6 (1.6/1.8)	8.7 (3.5/7.3)	1 (4.8/3.5)	29.9 (2.4/1.9)	68.4 (3.1/7)	5318.1 (0/4.8)
Munessa-Shashamene Forest	TM	1192 (15.3/56.6)	1203.5 (3.8/12.9)	10.1 (5.4/17.5)	17 (3.2/5.2)	23.8 (2.7/6.9)	13.7 (3.8/21.3)	0.8 (10/25.5)	13.1 (4.2/13.5)	42.9 (10.7/37.1)	5268.6 (0/5)
Paracou	TAr	3025.2 (17.3/64.6)	1246.2 (1.9/16.7)	22.5 (2.2/0.9)	26.3 (1.9/2.2)	30.1 (1.6/3.9)	7.6 (0/16.3)	0.9 (4.2/20.2)	29.5 (1.6/1.9)	58.7 (1.7/24.3)	5291.3 (0/3.8)
Pinkwae	TAwb	998.1 (22.2/64.7)	1255 (2.1/11.7)	24.1 (2/2.2)	27.5 (1.7/3.7)	30.9 (1.4/5.2)	6.8 (2.9/18.7)	0.7 (12.3/26.3)	27.7 (2.2/3.2)	73.6 (1.9/14.5)	5287.1 (0/4)
RBSF	TM	973.9 (35.2/65.6)	1439.9 (4.8/12)	4.7 (10.8/19.4)	13.1 (3.8/3)	21.7 (2.3/2.6)	17 (0.8/7.8)	0.5 (17/50)	11.4 (5.3/6.1)	75 (1.9/10)	5311 (0/6.4)
RFC	TAwa	1696.7 (13.7/70.8)	1184.6 (7.4/15.4)	22.3 (3.3/2.5)	27.3 (1.6/2.8)	32.4 (1.9/4.8)	10.1 (10.2/17.7)	0.9 (4/19.6)	27.6 (3.1/6.9)	82 (7.8/10)	5268 (0/5.1)
Rio Cachoiera	SCf	1526.6 (18.4/27)	913.4 (3/24.7)	12.6 (5.8/26.9)	18.1 (3.6/16.1)	23.7 (2.5/10.5)	11.1 (3/7.8)	1 (3.6/1.7)	16.5 (4.2/18.1)	78.1 (2.1/6.8)	4884.2 (0/24.7)
Selangor plantation	TAr	2840.3 (16.7/29.7)	1065.9 (3/8.4)	20.3 (3/1.7)	24.7 (2.1/1.5)	29.1 (1.5/2.1)	8.7 (3.7/8)	1 (1.8/1.5)	27.4 (2.6/2.6)	72.7 (2.6/10.6)	5304.8 (0/3.2)
SERS	TAwb	1558.7 (12.6/81)	1284.1 (2.7/14.7)	21.6 (2.5/10.9)	26.7 (1.7/6.5)	31.9 (1.4/5.5)	10.2 (4/21.7)	0.7 (5.7/37.8)	26.9 (2.1/14.8)	59.5 (4.4/34.2)	5147.3 (0/10.5)
Tapajos	TAr	1958.8 (19.9/63.6)	1126.8 (1.4/18.1)	21.8 (1.6/2.1)	26.5 (1.4/2.3)	31.2 (1.2/3.4)	9.4 (1.4/11.4)	0.9 (6.4/17.8)	29.7 (2/1.4)	75.5 (0.3/18.4)	5315.2 (0/5.7)
Tinte Bepo	TAr	1246.7 (15.5/61)	1123 (1.6/15.9)	21.8 (1.8/5.8)	26.5 (1.4/5.4)	31.3 (1.4/6.7)	9.5 (2.9/20.4)	0.8 (5.3/20.4)	25.1 (2.6/10)	75.7 (1.6/16.4)	5273.1 (0/4.8)
ZF-2	TAr	2232 (12.5/46.6)	1161.1 (1.9/16)	23.2 (1.7/1.1)	27.4 (1.3/1.8)	31.6 (1.3/2.7)	8.4 (4.5/9.7)	0.9 (5.5/11.2)	30.3 (2/2.3)	82.2 (1/13.6)	5316 (0/5.5)

Descriptive statistics of climate variables: Annual mean; coefficient of variation of annual means (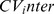) and coefficient of variation of monthly values (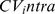) of precipitation (pre); potential evapotranspiration (pet); minimal, mean and maximal temperatures (respectively tmn, tmp and tmx); daily temperature amplitude (dtr); soil water content (swc); vapour pressure (vap); cloud cover (cld); and extraterrestrial solar radiation (sol). Coefficients of variations (standard deviation divided by the mean of the variable) of monthly climate time series are computed over the period of the CRU data (1901–2009). *^a^* Globale Ecological Zone defined by FAO [55]; TAr: Tropical rainforest; TAwa: Tropical moist deciduous forest; TAwb: Tropical dry forest; TM: Tropical mountain system; and SCf: Subtropical humid forest.

**Figure 2:**
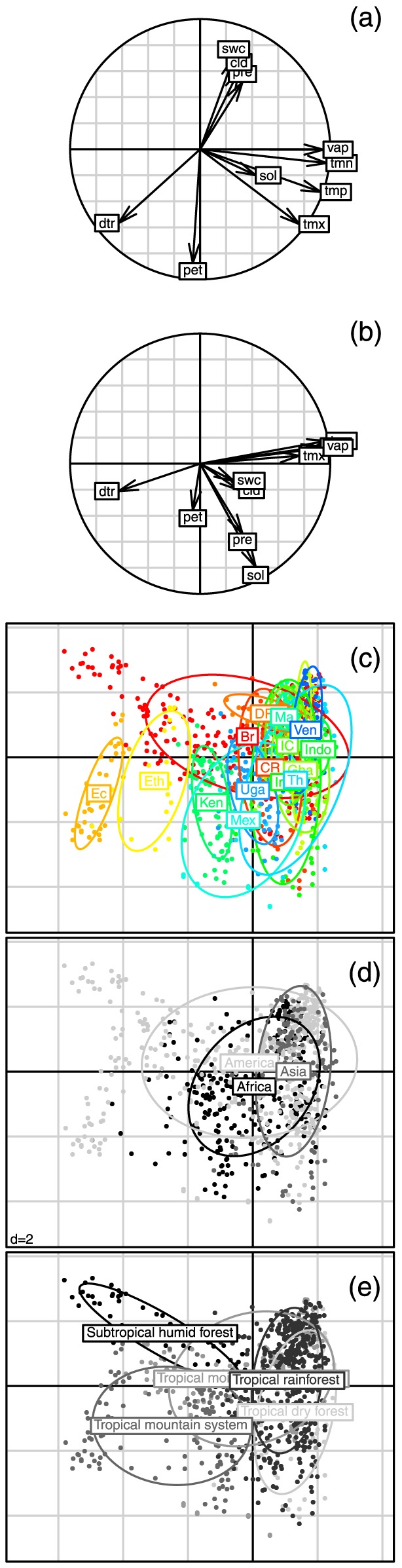
Principal component analysis of the climate variables. *cld*: cloud cover; *pre*: precipitation; *sol*: extraterrestrial solar radiation; *tmp*, *tmn* and *tmx* are respectively the daily mean, minimal and maximal temperatures; *dtr*: temperature amplitude; *vap*: vapour pressure; *pet*: potential evapotranspiration; and *swc*: relative soil water content. (a) correlation circle of axis 1 and 2; (b) correlation circle of axis 1 and 3; (c) projection of the country classes on the pca axis, India (Indi), Uganda (Uga), Brazil (Bra), Mexico (Mex), Costa Rica (CR), Kenya (Ken), Ivory Coast (IC), DRC (DRC), Indonesia (Indo), Ethiopia (Eth), French Guiana (FG), Ghana (Gha), Ecuador (Ec), Venezuela (Ven), Malaysia (Ma), Thailand (Th); (d) projection of the continental classes on the pca axis; and (e) projection of the global ecological zones on the pca axis (i.e. Tropical rainforest, Tropical moist deciduous forest, Tropical dry forest, Tropical mountain system and Subtropical humid forest). Note that axis 1 and 2 explain 41.70% and 28.12% of the total variation respectively, (a). The third axis explained 11.30% of the variance and was linked negatively to *sol* and *pre*, (b).

Three major climate gradients are represented in the Principal component analysis (PCA)(Figure 2). The first axis (x-axis in Figure 2a) represents a gradient of increasing temperature, that is highly correlated with vapour pressure. Sites close to the equator that are located at high altitudes are on the left of this axis, with sites far from the equator experiencing a cold season (Figure 2e). The second axis (y-axis in Figure 2a) has a strong contribution from variables that reflect water availability. The third axis (y-axis in Figure 2b) appears to be related to solar radiation. Ordination of the continental location of the studied sites on the PCA axis (Figure 2d) revealed that sites in Asia were slightly warmer and had higher vapour pressures than those in America and Africa. Ordination of the studied countries on the PCA axis (Figure 2c) reveals that some countries are separated from others because of climate: from cold (Ecuador) to warm (Indonesia) and from dry tropical (Mexico) to wet tropical (Malaysia)(Figure 2e). In the following analysis, we kept all the climate variables as we have a strong physiological assumption of their effect on tree growth (Table 1).

### Tree growth descriptive analysis

**Figure 3:**
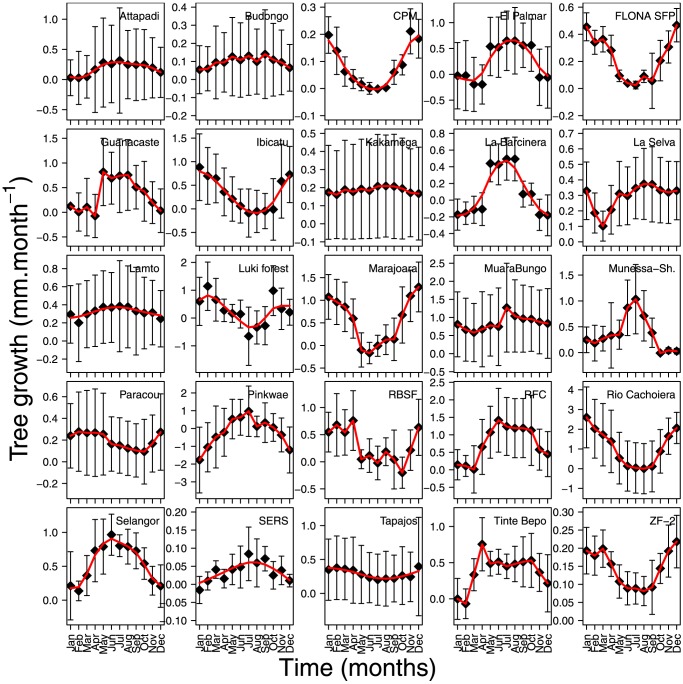
Evolution of mean monthly tree growth values and standard deviation for the studied forest sites. The red line represents a cubic smoothing spline.

We cannot reject the null hypothesis that there is no spatial autocorrelation present in mean tree growth observations between sites at alpha  =  0.05 (Moran's I observed = 0.0102, expected =  −0.042, SD 0.123, p.value  =  0.672). Tree diameter growth shows a significant intra-annual seasonality at all the studied sites, even sites with mean annual precipitation rates close to 4000 mm (La Selva, Costa Rica)(Table 5 and Figure 3).

**Table 5:** Seasonality of mean monthly tree growth with cosinor analysis for the studied sites.

Site	Amplitude	Phase	Low phase	p value
Attapadi	0.129	Month = 7.6	Month = 1.6	<0.05
Budongo	0.031	Month = 7.3	Month = 1.3	<0.05
CPM	0.104	Month = 12.2	Month = 6.2	<0.05
El Palmar	0.437	Month = 7.7	Month = 1.7	<0.05
FLONA SFP	0.213	Month = 1.1	Month = 7.1	<0.05
Guanacaste	0.396	Month = 7.3	Month = 1.3	<0.05
Ibicatu	0.470	Month = 1.5	Month = 7.5	<0.05
Kakamega	0.019	Month = 7.5	Month = 1.5	<0.05
La Barcinera	0.361	Month = 6.8	Month = 12.8	<0.05
La Selva	0.092	Month = 9	Month = 3	<0.05
Lamto	0.067	Month = 6.9	Month = 12.9	<0.05
Luki forest	0.535	Month = 1.4	Month = 7.4	<0.05
Marajoara	0.684	Month = 12.6	Month = 6.6	<0.05
Muara Bungo	0.211	Month = 8.6	Month = 2.6	<0.05
Munessa-Shashamene Forest	0.380	Month = 6.4	Month = 12.4	<0.05
Paracou	0.091	Month = 2.6	Month = 8.6	<0.05
Pinkwae	1.033	Month = 7	Month = 13	<0.05
RBSF	0.361	Month = 2.2	Month = 8.2	<0.05
RFC	0.645	Month = 7.6	Month = 1.6	<0.05
Rio Cachoiera	1.235	Month = 1.3	Month = 7.3	<0.05
Selangor plantation	0.380	Month = 6.7	Month = 12.7	<0.05
SERS	0.033	Month = 7.4	Month = 1.4	<0.05
Tapajos	0.089	Month = 1.9	Month = 7.9	<0.05
Tinte Bepo	0.223	Month = 6.9	Month = 12.9	<0.05
ZF-2	0.066	Month = 1.1	Month = 7.1	<0.05

Month  =  1 corresponds to January. The amplitude is the difference between the higher and lower points in mm.month^−1^ of the sinusoid fitted in the cosinor analysis. The phase and the low phase are, respectively, the month with the highest/lowest tree growth value according to the sinusoid fitted in the cosinor analysis. A 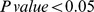 indicates that the statistically significant existence of a seasonal pattern cannot be rejected.

### Climate effects on tree growth

**Figure 4:**
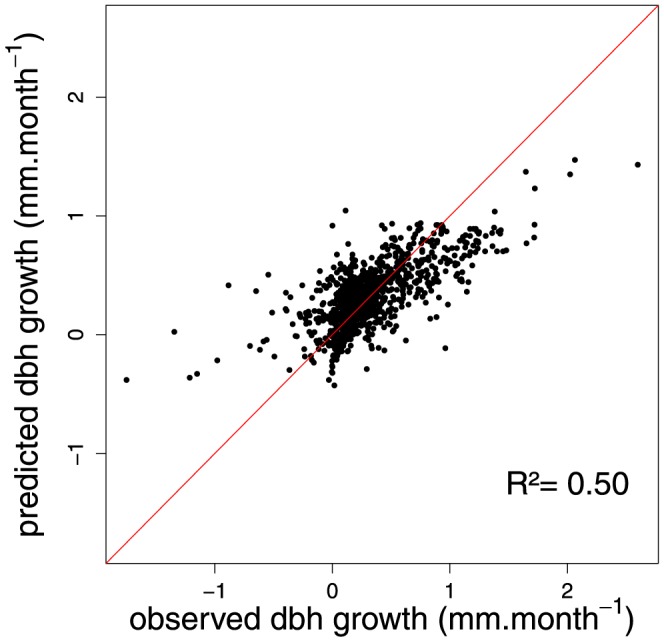
Observed versus predicted diameter at breast height (dbh) growth under the model m*_BIC_*. The red line is the identity line y  =  x. Note that the model underestimated the diameter growth above 1 mm.month^−1^.

**Figure 5:**
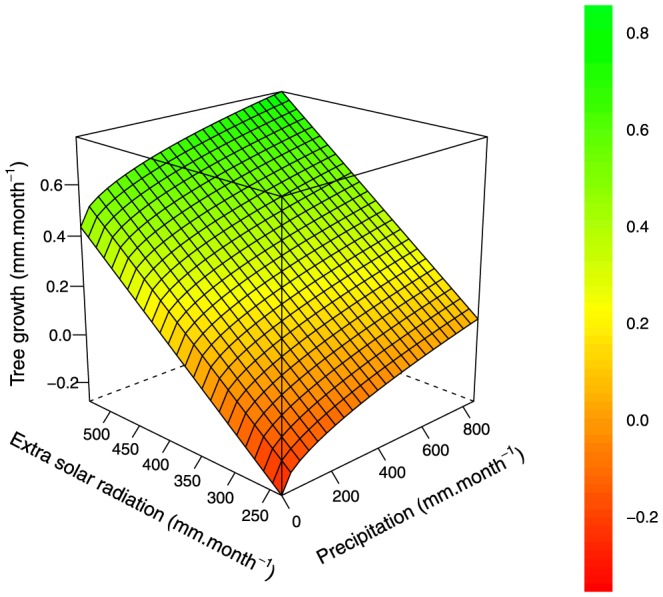
Predicted diameter at breast height (dbh) growth under the model *m_BIC_* and relationship with precipitation and extra solar radiation. Predictions were computed using *pre*, *sol*, mean *dtr*, mean *swc* and mean of the random effect. The extra solar radiation unit is equivalent of evaporation in mm.month^−1^, 1 mm.month^−1^ equivalent of evaporation  =  2.45 MJ.m^−2^.month^−1^.

More than 28.69% of the observed seasonal variation in tree growth may be imputable to the site effect (reference model m_0_, Table 6), while climate variables alone explain a maximum of 19.82% (squared root monthly precipitation). The variables *sol*, *cld*, *vap*, *dtr*, *tmn* and *swc* explained between 9.65 and 16.30% of the climate effect, while *tmp*, *pet* and *tmx* explained less than 2.13%. The selection procedure, which used the BIC criterion, kept four climatic variables (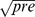, *sol*, *dtr*, *swc*) in the final multivariate model *m_BIC_* (Table 6). These four climate variables together explained 29.79% of the total observed monthly tree growth variation. The parameter values for the fixed effect in the univariate analysis indicate the direction of the relation between the climate variables and tree growth (Table 6). Among the four selected variables, *pre* (0.03), *swc* (0.58) and *sol* (0.09) have a positive link with tree growth and *dtr* (−0.08) has a negative link (Table 6). The obtained root mean squared error of prediction (RMSEP) was slightly below the mean value of observed monthly growth in diameter (mean growth  =  0.325 mm.month^−1^, RMSEP = 0.279 mm.month^−1^). In general, the model underestimated growth when it was above 1 mm.month^−1^ (Figure 4). There was no significant correlation between the selected variable and the residuals of the model m*_BIC_* (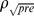  =  4.6×10^−6^, pvalue > 0.99; *ρ_sol_*  =  1.8×10^−5^, pvalue > 0.99; *ρ_dtr_*  =  1.9×10^−4^, pvalue > 0.99; *ρ_swc_*  =  6.1×10^−6^, pvalue > 0.99). Tree growth values increase linearly with extrasolar radiations (Figure 5), and a strong increase in tree growth occurred between 0 and 200 *mm.month* ^−1^ of precipitation; above these values, the increase in tree growth was less pronounced and had a linear shape.

**Table 6:** Model parameters, standard errors, t values and posterior densities of the univariate (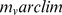) and complete (*m_BIC_*) analyses.

Model	Parameter	Effect	Estimates	Std. Error	t value	Variance	Std. Dev.	MCMC median	MCMC mean	HPD95 lower	HPD95 upper	pMCMC	Pr(>|t|)	RMSEP	BIC	R2 fixed	R2
*m_BIC_*	site.names	random				0.0355	0.1885	0.1577	0.1596	0.1184	0.2038			0.2787	412.4698	29.79	50.25
	Residual	random				0.0800	0.2829	0.2845	0.2846	0.2713	0.2975						
	(Intercept)	fixed	−0.7247	0.1635	−4.4336				−0.7492	−1.0585	−0.4339	0.0001	0.0000				
	I(pre∧0.5)	fixed	0.0118	0.0027	4.4017				0.0120	0.0067	0.0171	0.0001	0.0000				
	sol_m	fixed	0.0024	0.0002	10.3592				0.0024	0.0019	0.0028	0.0001	0.0000				
	dtr	fixed	−0.0300	0.0078	−3.8291				−0.0283	−0.0430	−0.0139	0.0002	0.0001				
	swc	fixed	0.2426	0.0674	3.5999				0.2492	0.1196	0.3817	0.0002	0.0003				
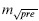	site.names	random				0.0346	0.1861	0.1588	0.1608	0.1192	0.2057			0.2983	515.5249	19.82	42.99
	Residual	random				0.0914	0.3023	0.3038	0.3040	0.2901	0.3184						
	(Intercept)	fixed	−0.0158	0.0394	−0.4006				−0.0157	−0.0860	0.0562	0.6666	0.6888				
	I(pre∧0.5)	fixed	0.0303	0.0020	15.1909				0.0303	0.0264	0.0343	0.0001	0.0000				
m*_sol_*	site.names	random				0.0414	0.2034	0.1695	0.1715	0.1296	0.2177			0.3043	556.2737	16.30	40.67
	Residual	random				0.0951	0.3084	0.3103	0.3104	0.2956	0.3245						
	(Intercept)	fixed	−1.0027	0.1042	−9.6261				−0.9997	−1.1959	−0.7884	0.0001	0.0000				
	sol_m	fixed	0.0030	0.0002	13.4849				0.0030	0.0026	0.0035	0.0001	0.0000				
m*_cld_*	site.names	random				0.0333	0.1825	0.1576	0.1594	0.1183	0.2040			0.3065	563.8953	15.56	39.81
	Residual	random				0.0965	0.3106	0.3120	0.3122	0.2975	0.3264						
	(Intercept)	fixed	−0.4837	0.0691	−6.9952				−0.4766	−0.6100	−0.3444	0.0001	0.0000				
	cld	fixed	0.0115	0.0009	13.1090				0.0114	0.0097	0.0132	0.0001	0.0000				
M*_pre_*	site.names	random				0.0345	0.1856	0.1601	0.1621	0.1193	0.2073			0.3078	571.9796	14.82	39.34
	Residual	random				0.0972	0.3118	0.3133	0.3135	0.2992	0.3284						
	(Intercept)	fixed	0.1542	0.0352	4.3855				0.1542	0.0933	0.2184	0.0001	0.0000				
	pre	fixed	0.0011	0.0001	12.7430				0.0011	0.0009	0.0013	0.0001	0.0000				
m*_dtr_*	site.names	random				0.0705	0.2655	0.1957	0.1982	0.1523	0.2514			0.3060	579.4892	14.60	40.01
	Residual	random				0.0962	0.3102	0.3134	0.3136	0.2998	0.3290						
	(Intercept)	fixed	1.2160	0.0842	14.4368				1.1559	0.9919	1.3130	0.0001	0.0000				
	dtr	fixed	−0.0840	0.0066	−12.655				−0.0783	−0.0925	−0.0650	0.0001	0.0000				
m*_vap_*	site.names	random				0.1335	0.3653	0.2128	0.2152	0.1607	0.2740			0.3063	596.8096	13.87	39.89
	Residual	random				0.0964	0.3105	0.3167	0.3169	0.3017	0.3314						
	(Intercept)	fixed	−1.1021	0.1306	−8.4356				−0.8118	−1.0723	−0.5445	0.0001	0.0000				
	vap	fixed	0.0593	0.0048	12.3119				0.0472	0.0368	0.0579	0.0001	0.0000				
m*_swc_*	site.names	random				0.0319	0.1785	0.1564	0.1582	0.1146	0.2039			0.3174	625.9566	9.77	35.49
	Residual	random				0.1034	0.3215	0.3229	0.3230	0.3082	0.3382						
	(Intercept)	fixed	−0.1696	0.0580	−2.9216				−0.1715	−0.2826	−0.0609	0.0038	0.0036				
	swc	fixed	0.5807	0.0578	10.0435				0.5837	0.4723	0.7025	0.0001	0.0000				
m*_tmn_*	site.names	random				0.0944	0.3072	0.2038	0.2063	0.1523	0.2625			0.3143	635.0120	9.65	36.71
	Residual	random				0.1015	0.3186	0.3231	0.3233	0.3085	0.3387						
	(Intercept)	fixed	−0.8685	0.1291	−6.7262				−0.6662	−0.9239	−0.4010	0.0001	0.0000				
	tmn	fixed	0.0623	0.0062	10.0385				0.0517	0.0393	0.0656	0.0001	0.0000				
m*_tmp_*	site.names	random				0.0396	0.1989	0.1693	0.1716	0.1238	0.2187			0.3299	701.8122	2.13	30.27
	Residual	random				0.1117	0.3343	0.3359	0.3361	0.3210	0.3522						
	(Intercept)	fixed	−0.3704	0.1566	−2.3648				−0.3351	−0.6345	−0.0317	0.0266	0.0182				
	tmp	fixed	0.0283	0.0063	4.5113				0.0269	0.0146	0.0388	0.0001	0.0000				
m*_pet_*	site.names	random				0.0329	0.1814	0.1600	0.1620	0.1178	0.2075			0.3320	709.0369	1.40	29.38
	Residual	random				0.1132	0.3364	0.3377	0.3379	0.3219	0.3532						
	(Intercept)	fixed	0.5525	0.0724	7.6338				0.5589	0.4147	0.6951	0.0001	0.0000				
	pet	fixed	−0.0023	0.0006	−3.6135				−0.0023	−0.0036	−0.0011	0.0002	0.0003				
m*_tmx_*	site.names	random				0.0400	0.2000	0.1717	0.1738	0.1278	0.2217			0.3334	721.5133	0.05	28.78
	Residual	random				0.1141	0.3378	0.3395	0.3396	0.3234	0.3550						
	(Intercept)	fixed	0.4356	0.1755	2.4825				0.4047	0.0543	0.7394	0.0190	0.0132				
	tmx	fixed	−0.0039	0.0058	−0.6821				−0.0029	−0.0141	0.0086	0.6180	0.4954				
m_0_	site.names	random				0.0385	0.1962	0.1696	0.1717	0.1270	0.2214			0.3337	715.0984	0.00	28.69
	Residual	random				0.1142	0.3379	0.3395	0.3396	0.3240	0.3551						
	(Intercept)	fixed	0.3184	0.0347	9.1815				0.3187	0.2571	0.3784	0.0001	0.0000				

Model parameters, standard errors and t-values. The parameter values of posterior parameters densities (MCMC median and MCMC mean) and their 95% confidence intervals (Highest posterior density at 95% [HPD95] lower and upper) and associated p-value are estimated from a distribution of 10000 parameter samples computed with Monte Carlo Markov Chains from the model parameters. Goodness-of-fit criterion of the growth models are root mean square error of prediction (RMSEP), Bayesian Information Criterion (BIC), R^2^ of the fixed part of the model (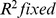) and R^2^ of the complete model (fixed plus random part).

## Discussion

Our meta-analysis provides the first overview of climate effects on tree growth variations at a seasonal time scale for tropical forest areas. Tree growth reveals a strong intra-annual seasonality at all sites (Table 5), including seasonally dry sites and wet tropical forests. We found a common climatic signal explaining 29.79% of the observed seasonality in forest growth (Table 6). Precipitation (19.82%) and extraterrestrial solar radiation (16.30%) were the major climate drivers. The tree growth average per site (i.e. the random site effect) explained 28.69% of overall growth variation, and a substantial fraction of variation (50%) in growth remained unexplained with our modeling framework. We have to acknowledge than our sample of 3412 trees could not be used to fully reproduce the complete behavior of all the tropical forests, however our analysis demonstrated that a common signal in the climate determinism of tree growth seasonality is observed at the pan-tropical scale.

### Climate effect on tree growth

In this study, precipitation was found to be the major seasonal driver of tree growth. Precipitation strongly impacts tree growth, as directly observed in seasonal and unseasonal tropical climates [19], [35], [56]–[59] and as deduced from experimental forest droughts [60], [61]. The prominence of precipitation as a predictor of forest growth is slightly different from previous studies [62]; in French Guiana, we found that soil water availability was the main determinant of diameter growth, i.e. better than rainfall. Of course, both variables are strongly correlated (Figure 2), but this difference could be explained by some sites where relative soil water availability *swc* was at its maximum throughout the year despite marked precipitation seasonality (e.g. La Selva, CPM, Muara Bungo, Rio Cachoiera and Selangor). At the same time, the importance of solar radiation, *sol*, in the complete model (*m_BIC_*) reflects the obvious role of light in shaping tree growth (Figure 5). Solar radiation is directly linked to PPFD (Photosynthetic Photon Flux Density), which in turn drives carbon uptake and plant growth [29]. Some authors support the hypothesis that increasing surface solar radiation contributes to the increasing forest growth rate over the Amazon [63], [64]. It must be noted that the extraterrestrial solar radiation we used has a value above the real solar radiation value reaching the forest surface. Indeed, *sol* was computed as a monthly mean over 50 years and does not account for local cloud cover or aerosol radiation absorption.

Investigating the effects of temperature on the physiology of tropical forest trees [31], [65] is of primary importance today given the temperature increases expected over the next century [5], [7]. Some authors suggest that tropical trees are more sensitive to temperatures than other trees because (i) they live at or close to the highest annual average temperatures on Earth, and (ii) tropical species naturally encounter limited variation in temperatures (<4°C over 20° of latitude) [32]. Our results suggest that temperature variations are of secondary importance in shaping tropical tree seasonal growth; nevertheless, they do play a role. Minimal temperature was slightly positively correlated with tree growth, whereas maximal temperature had no effect. This positive relation between tree growth and temperature is not consistent with previous observations in Costa Rica [35], [66], where an increase in night-time temperatures had a negative effect on tree growth, and in East Africa [19], where maximum temperatures had a negative effect on tree growth. Such patterns were not found at our global working scale.

### Tree growth variability in time and space

Our results suggest that most tropical trees experience seasonal cycling growth even in extremely wet environments, as already reported at la Selva [35]. On a pantropical scale, we showed that secondary growth was higher during the wet season; tree growth increased with precipitation and relative soil water content (Table 6). This result is not consistent with other studies that have shown that tropical forests are able to maintain or even to increase their productivity during the dry season [67]–[70]. This suggests different uses of carbohydrates. Some studies have reported that evergreen species in seasonally dry environments accumulate carbohydrates during the dry season because photosynthesis continues while wood production ceases [71] and that deciduous species accumulate carbohydrates at the onset of the dry season to support respiration costs when they are leafless [72], [73]. Some studies relied on the Enhanced Vegetation Index (EVI, an index of canopy photosynthetic capacity [30]) to highlight changes in forest phenology driven by the solar cycle [74], [75]. In the same way, a variation in the EVI has been observed for the entire Amazon region [30], and the link between phenology and the solar cycle has also been reported for Terra Firme forests [76]. The variability in tree growth at a seasonal scale is likely driven by climate seasonality and dependent on the seasonal allocation of carbohydrates to processes other than tree diameter growth, such as leaf and root production or respiration.

Stem shrinkage during dry periods may be an important limitation of this work [77]–[79], e.g. the negative value of mean annual tree growth at Pinkwae. Other negative monthly growth values exist at almost all the study sites. In a tropical forest in Ethiopia experiencing a strong seasonality, high-resolution electronic dendrometers have been combined with wood anatomy investigation to describe cambial growth dynamics [80]. These authors concluded that water scarcity during the long dry season induced cambial dormancy. Furthermore, after the onset of the rainy season, (i) bark swelling started quite synchronously among trees; (ii) bark swelling was maximum after few rainy days; and (iii) evergreen trees were able to quickly initiate wood formation. Recently at the Paracou forest site, some authors have showed that biomass increments were highly correlated between the first and the last quantile of trunk bark thickness and between the first and the last quantile of trunk bark density, suggesting that secondary growth is driven by cambial activity [75].

In this study, we focused on seasonal variation of tree growth, but the inter-site variance must be studied to build a full predictive model. At our pantropical working scale, there was no evident spatial auto-correlation. The site effect, i.e. the average growth of trees at a given site, is likely shaped by several additional environmental variables, such as soil fertility, forest floristic composition and competition for light and nutrients [81]–[83]. These effects were included in the model through the random site effect 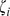, and we assumed that these effects were constant over the study period. We also did not consider the different ontogenetic stages of trees and the ontogenetic growth trajectory that depends on complex environmental changes that may have occurred during the census period [84]. Recently, a study using LiDAR and a four-year diameter growth census demonstrated that variation in canopy metrics appeared to be essential to predict biomass growth [85]. Clearly, remote sensing methods, whether radar, LiDAR or optical [86], can help us to derive stand variables, especially canopy height, and therefore can be used as valuable additional predictors to monitor forest growth over large tropical areas.

### Tropical tree growth under climate change

Globally, current IPCC scenarios predict an intensification of the dry season in tropical areas during the 21st century [7]. Amongst climate variables, our results highlighted the predominant role of precipitation in shaping seasonal forest growth. If a global rainfall reduction is confirmed in the future, it can be expected that tree growth will be affected (Figure 5). Climate change is not the only possible cause for reductions in precipitation; deforestation could lead to reductions in precipitation as the air passages over forests increase tropical rainfall intensity [4]. Solar radiation appeared almost as important as rainfall. Cloud reduction due to drier dry seasons, may subsequently improve tree growth. Our results suggest that, with the global changes observed, forest productivity due to tropical tree growth will encounter modifications due to change in climate seasonality.
